# The effect of capnography on the incidence of hypoxia during sedation for EGD and colonoscopy in mildly obese patients: a randomized, controlled study

**DOI:** 10.1186/s12871-023-02151-8

**Published:** 2023-05-31

**Authors:** Yingjie Wang, Fang Liu, Yuan Zhang, Xiaomei Yang, Jianbo Wu

**Affiliations:** 1 Department of Anesthesiology, Qilu Hospital of Shandong University, Jinan, 250012 China; 2 School of Medicine, Cheeloo College of Medicine, Shandong University, Jinan, 250012 China; 3 Department of Cardiology, the Key Laboratory of Cardiovascular Remodeling and Function Research, Chinese Ministry of Education, Chinese National Health Commission and Chinese Academy of Medical Sciences, the State and Shandong Province Joint Key Laboratory of Translational Cardiovascular Medicine, Qilu Hospital, Cheeloo College of Medicine, Shandong University, Jinan, 250012 China; 4 Department of Anesthesiology and Perioperative Medicine, Qilu Hospital Dezhou Hospital, Shandong University, Dezhou, 253000 China

**Keywords:** Anesthetic i.v., Adverse effects, Capnography, Outcomes, EGD and Colonoscopy

## Abstract

**Background:**

By continually monitoring end-tidal carbon dioxide concentrations, capnography can detect abnormal ventilation or apnoea early. This randomized, controlled study explored the effect of early intervention with capnography on the incidence of hypoxia in mildly obese patients undergoing sedation for esophagogastroduodenoscopy (EGD) and colonoscopy.

**Methods:**

This is a single-center, randomized, single-blind, parallel-assignment, controlled trial. Mildly obese patients (28 kg/m^2^ ≤ BMI < 40 kg/m^2^) undergoing sedation for EGD and colonoscopy were randomly assigned to either the standard or capnography group. Standard cardiopulmonary monitoring equipment was used in both groups, and additional capnography was performed in the capnography group. In the event of inadequate alveolar ventilation during sedation, five interventions were administered in sequence (a-e) : a: increasing oxygen flow (5 L/min); b: a chin lift or jaw thrust maneuver; c: placement of the nasopharyngeal airway and chin lift; d: mask positive-pressure ventilation, and e: ventilator-assisted ventilation with tube insertion. The primary outcome was the incidence of hypoxia (SpO_2_ < 90%, ≥ 10 s) in each group. The secondary outcomes included the incidence of severe hypoxia (SpO_2_ ≤ 85%), subclinical respiratory depression (90% ≤ SpO_2_ < 95%), interventions, minimum SpO_2_ during operation, patient satisfaction, endoscopist satisfaction, and other adverse events of anesthesia sedation.

**Results:**

228 patients were included (capnography group = 112; standard group = 113; three patients were excluded) in this study. The incidence of hypoxia was significantly lower in the capnography group than in the standard group (13.4% vs. 30.1%, *P* = 0.002). Subclinical respiratory depression in the capnography group was higher than that of the standard group (30.4% vs. 17.7%, *P* = 0.026). There was only a 5.4% incidence of severe hypoxia in the capnography group compared with 14.2% in the standard group (*P* = 0.026). During sedation, 96 and 34 individuals in the capnography and standard groups, respectively, underwent the intervention. There was a statistically significant difference (*P* < 0.0001) in the number of the last intraoperative intervention between the two groups ( a:47 vs. 1, b:46 vs. 26, c:2 vs. 5, d:1 vs. 2, e:0 vs. 0 ). No significant differences were found between the two groups in terms of minimum SpO_2_ during operation, patient satisfaction, or endoscopist satisfaction rating. There was no statistically significant difference in adverse events of anesthesia sedation between the two groups.

**Conclusion:**

Capnography during sedation for EGD and colonoscopy allows for the detection of apnea and altered breathing patterns in mildly obese patients before SpO_2_ is reduced. Effective intervention measures are given to patients within this time frame, which reduces the incidence of hypoxia and severe hypoxia in patients.

**Trial registration:**

Ethical approval was granted by the Medical Ethics Committee (Chairperson Professor Tian Hui) of Qilu Hospital, Shandong University ((Ke) Lun Audit 2021 (186)) on 15/07/2021. The study was registered (https://www.chictr.org.cn) on 23/10/2021(ChiCTR2100052234). Designed and reported using CONSORT statements.

**Supplementary Information:**

The online version contains supplementary material available at 10.1186/s12871-023-02151-8.

## Background

Sedation for endoscopy is becoming increasingly common as a means of diagnosing and treating gastrointestinal diseases, increasing patient comfort and compliance, and improving the efficiency of disease diagnosis and treatment [1]. However, sedation/anesthesia has high risks, and some complications can cause serious consequences or even death [2, 3], such as respiratory depression and hypoxia [4, 5].

In obese patients, the oxygen reserve capacity is poor, and a few patients have sleep apnea syndrome, which can easily cause posterior tongue drop in general intravenous anesthesia and lead to upper airway obstruction, respiratory depression, SpO_2_ drop, choking and other complications [6–8]. Recent studies have shown that body mass index (BMI) is an independent risk factor for sedation-related adverse events [9–12]. Moreover, severe hypoxia often requires suspension of endoscopy for mask ventilation or tracheal intubation-assisted ventilation, and prolonged hypoxia can also lead to myocardial ischemia, malignant arrhythmias, permanent neurological damage, or even death [4, 5]. Therefore, it is essential to prevent hypoxia in mildly obese patients undergoing sedation for EGD and colonoscopy.

Using pulse oximetry alone can significantly delay the detection of apnea or hypoventilation, especially if the patient receives supplemental oxygen [13, 14]. P_ET_CO_2_ values and waveforms can provide a more accurate picture of a patient’s pulmonary ventilation status and guide the early implementation of respiratory interventions. As a monitoring technique, capnography is widely used in clinical practice because of its high sensitivity and noninvasiveness.

Currently, there is no consensus on whether capnography can effectively reduce the occurrence of hypoxia during endoscopy. Beltz et al. [15–17]believe that capnography can reduce the occurrence of hypoxia in patients during the examination. However, some researchers believe that capnography does not increase patient satisfaction and safety but instead increases the cost of the test [18].

In this study, we explored whether intra-operative capnography could effectively reduce the incidence of hypoxia in patients undergoing sedation for EGD and colonoscopy, limiting the subjects to mildly obese patients who are more prone to intra-operative hypoxia.

## Methods

### Study design

This single-center, prospective, randomized, controlled trial was conducted from November 2021 to July 2022 at Qilu Hospital of Shandong University in Jinan, China. Ethical approval was granted by the Medical Ethics Committee (Chairperson Professor Tian Hui) of Qilu Hospital, Shandong University ((Ke) Lun Audit 2021 (186)) on 15/07/2021. The study was registered on 23/10/2021 (https://www.chictr.org.cn). We designed and reported this study using CONSORT statements. We obtained written, informed consent from the patient, his or her next of kin, or a legal representative.

### Study population

Inclusion criteria: (1) American Society of Anesthesiologists classification (ASA) class I or II; (2) age 18–65 years; (3) 28 kg/m^2^ ≤ BMI < 40 kg/m^2^; (4) undergoing sedation for EGD and/or colonoscopy procedures, (5) informed consent from the patient or family. Exclusion criteria: (1) nasal bleeding, nasal mucosal injury, space-occupying lesions in the nasal cavity; (2) diagnosed heart disease (heart failure, angina pectoris, heart attack, arrhythmia); (3) diagnosed lung disease (asthma, bronchitis, COPD, pulmonary maculopathy, pulmonary embolism, lung cancer); (4) those with previous hypotension (systolic blood pressure ≤ 90 mmHg), bradycardia (heart rate < 50 beats/min), or hypoxemia (SaO_2_ < 90%); (5) presence of underlying disease requiring oxygen; (6) emergency surgery; (7) multiple trauma; (8) upper respiratory tract infection; (9) allergy to sedative drugs such as propofol or devices such as tape, and (10) disagreement to participate in this study. Post-inclusion exclusion criteria: (1) failure to complete endoscopy by sedation; (2) serious adverse events during the experiment forcing the patient to discontinue endoscopy; and (3) noncompliance with the protocol requirements.

### Randomization and blinding

Patients were randomly divided into a capnography group or a standard group in a 1:1 ratio using a random sequence of numbers generated through computerized randomization software. In the capnography group, the capnographic data of the patients were available for additional noninvasive assessment of ventilation. The capnographic data of patients assigned to the standard monitoring group were not visible because the carbon dioxide sampling port of the nasal cannula was kept closed; therefore, only the integrated pulse oximetric readout of the monitor was visible.

Investigators randomly assigned patients to the study groups by opening pre-generated, sequentially numbered, opaque, sealed envelopes. The patients, endoscopy unit staff, and endoscopists were blinded to the study arm assignments. Both groups used nasal cannulae with carbon dioxide collection devices in identical packaging and shapes that were connected to capnography devices. A subject could be unblinded in the event of a severe adverse event requiring hospitalization or prolonged hospitalization (disability, affecting the ability to work; life-threatening or death; causing congenital malformations) or another emergency. The sponsor, study director, and clinical monitor were notified before unblinding.

### Study procedure

Routine heart rate monitoring, blood pressure monitoring, and electrocardiography were performed on all patients. For the assessment of SpO_2_, the integrated pulse oximeter of the capnography device was used. A nasal cannula (Capnostream 20; Medtronic, Inc.) with an oral sampling port to accommodate mouth breathers provided 2 L/min oxygen and continuously sampled the CO_2_ content of both inspired and expired patient gas. The sampling line was connected to a portable bedside monitor (Capnostream 20; Medtronic, Inc.) that displayed a time-based capnogram, P_ET_CO_2_ (mmHg), the derived respiratory rate, and SpO_2_ by integrated pulse oximetry (Nellcor, Covidien, Boulder, CO, USA).

The patient was placed in the lateral position and pre-oxygenated by deep breathing with 2 L min^− 1^ oxygen. 5 or 7.5 µg of intravenous sufentanil (sufentanil citrate injection, 50 ug 1 mL^− 1^ampule^− 1^; Yichang Humanwell Pharmaceutical Co, Ltd, Yichang, China) was administered during the induction of anesthesia in both groups. After 3 min, sedation was induced by a 1.5 mg/kg intravenous injection of propofol (propofol injection long-chain triglyceride, 200 mg /20 Ml/ampule, Fresenius Kabi Deutschland, Bad Homburg, Germany), administered by hand at a rate of 0.5 ml/s. Sedation depth was evaluated according to the Modified Observer’s Assessment of Alertness/Sedation (MOAA/S) score [19] (Table S2 in Supplementary Appendix). According to our routine practice, deep sedation to a MOAA/S score of 0/1 was performed first to ensure the successful insertion of the endoscope tube and to decrease adverse responses to the insertion of the endoscope tube into the upper airway. During endoscopy, moderate sedation with a MOAA/S score of 2/3 was maintained with additional propofol (20–30 mg).

In the event of inadequate alveolar ventilation during sedation, the investigators administered the following five interventions sequentially until all respiratory-related parameters returned to normal, recording the means of the last intervention: a: increasing oxygen flow (5 L/min); b: a chin lift or jaw thrust maneuver; c: placement of the nasopharyngeal airway and a chin lift; d: mask positive-pressure ventilation, and e: ventilator-assisted ventilation with tube insertion. The division of labor among participants was fixed throughout the experiment, and the gastroenterologist performed the endoscopy.

After endoscopy completion, patients who can provide meaningful verbal responses and have stable vital signs were withdrawn from monitoring and transferred to the recovery room. Discharge was allowed when a post-anesthesia discharge scoring system (PADSS) (Table S3 in Supplementary Appendix) score of > 9 was met. When fully recovered, patient satisfaction of sedation was assessed through a numeric analog scale (Table S4 in Supplementary Appendix) before patients were discharged. Endoscopists were asked to rate their satisfaction of sedation on a numeric analog scale (1, min – 10, max) at the end of the procedure.

Adverse events were any instances where adverse symptoms and abnormal signs occurred after the application of the intervention, whether or not they were causally related to the trial. Any adverse events, including those provided voluntarily by the subject or identified through investigator inquiries and monitoring, were actively managed and closely followed until they resolved or stabilized. Adverse events of anesthesia sedation, including PONV, hypotension, bradycardia, and premature ventricular contractions occurred, were treated with ondansetron hydrochloride, atropine, noradrenaline, and lidocaine, respectively. (Table S5 in Supplementary Appendix)

### Study outcome

Inadequate alveolar ventilation during sedation included altered ventilation, apnoea, or decreased oxygen saturation. The capnographic criterion for apnoea was the absence of exhaled CO_2_; altered respiration was defined as a reduction in end-tidal CO_2_ by more than half from the baseline value; decreased oxygen saturation was defined as SpO_2_ < 90%, ≥ 10s.

The primary outcome was the incidence of hypoxia during sedation, defined as a decrease in SpO_2_ to ≤ 90%, ≥ 10s. The period of sedation was defined as the period from the start of the first medication administration until the discontinuation of electronic monitoring after the completion of the procedure.

The secondary outcomes included (i) incidence of subclinical respiratory depression (90% ≤ SpO_2_ < 95%) and severe hypoxia (SpO_2_ ≤ 85%); (ii) interventions; (iii) minimum SpO_2_ during operation; iv) patient and endoscopist satisfaction, v) adverse events of anesthesia sedation.

### Statistical analysis

The sample size was calculated using PASS software (version 11.0, NCSS, LLC, Kaysville, UT, United States). The Two Independent Proportions procedure was used. Test results indicate a 32% incidence of hypoxia in mildly obese patients under sedation procedure; thus, 32% of patients in the capnography group were expected to develop hypoxia. P1 and P2 were calculated based on the assumption that capnography would achieve a reduction in the incidence of hypoxia from 32 to 15%. Given an α = 0.05 and a power of 80%, it was estimated that 108 patients per group would be required for the study. Assuming a dropout rate of 5%, a total of 228 patients would be needed.

A blinded statistician performed statistical analysis using SPSS (version 25.0). Data are summarized as mean ± SD/median (IQR) for continuous data and as frequencies/percentages for categorical data. The standardized mean difference (SMD) was defined as the difference between the mean value of a covariate in one group and the corresponding mean value of a covariate in the other group, divided by the pooled standard deviation. When assessing the balance between groups, an SMD < 0.1 indicated a better balance and was considered as a small difference between the two groups. The incidence of hypoxia for the primary outcome was analyzed using Fisher’s exact test for categorical variables. Secondary outcomes were also analyzed with the use of Fisher’s exact test for categorical variables as well as Student’s t-test for continuous variables with a composite normal distribution. Statistical significance was set at *P* < 0.05.

## Results

### Intention-to-treat analysis

**Fig. 1:**
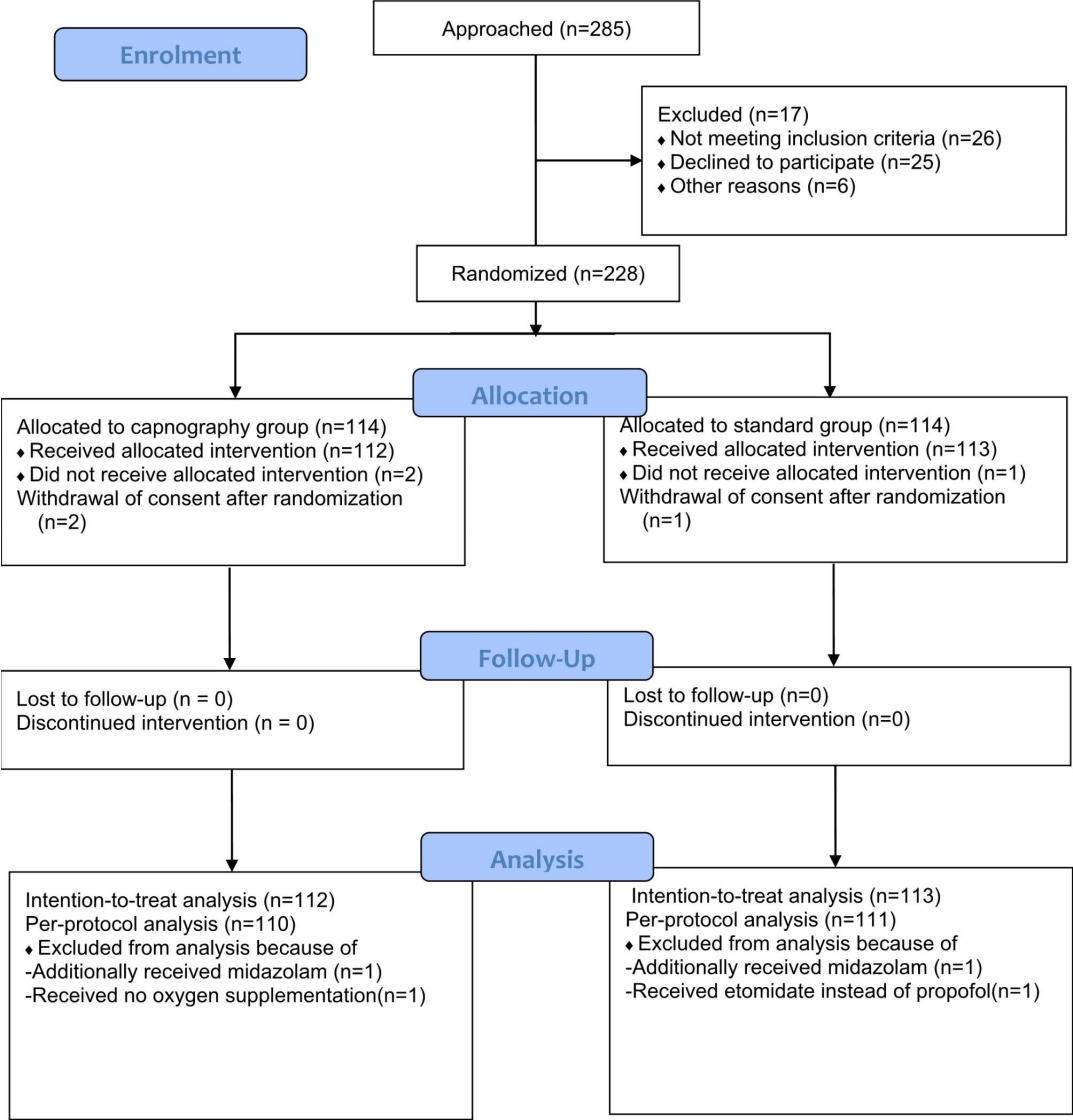
Randomization flowchart of the study population

From November 2021 to July 2022, 285 patients presenting with EGD and/or colonoscopy were screened for enrollment. Of these, 26 patients did not meet the eligibility criteria, 25 refused to participate, and six were excluded for other reasons (surgery was canceled; withdrawn by the treating physician). A total of 228 subjects underwent randomization. Three patients withdrew their consent after randomization. Thus, 225 patients were included in the intention-to-treat analysis (Fig. 1). There were minimal differences in baseline demographic, clinical, and surgical characteristics between the two groups of patients(SMD<0.1) Table 1).

**Table 1:** Comparison of the randomized groups on baseline variables

	Capnography (n = 112)	Standard (n = 113)	SMD
Age (years)	47.0 ± 10.2	47.3 ± 10.2	0.030
Sex, male	85(75.9)	88(77.9)	0.047
BMI, kg/m^2^	30.7 ± 2.3	30.8 ± 2.1	0.030
ASA class			0.096
I	87(77.7)	93(82.3)	
II	25(22.3)	20(17.7)	
History of smoking	43(38.4)	39(34.5)	0.081
History of alcohol consumption	41(36.6)	40(35.4)	0.025
Mallampati Class			0.087
I	36(32.1)	40(35.4)	
II	70(62.5)	70(61.9)	
III	6(5.4)	3(2.7)	
TMD < 6 cm	27(24.1)	24(21.2)	0.069
Micrognathia or retrognathia	16(14.3)	16(14.2)	0.004
STOP-BANG questionnaire score > 3分	34(30.4)	34(30.1)	0.006
Type of procedure			
EGD	41(36.6)	44(38.9)	
Colonoscopy	8(7.1)	14(12.4)	
Colonoscopy + EGD	63(56.3)	55(48.7)	
Procedure time, minutes	25.2 ± 10.7	25.2 ± 12.7	0.001
Induction dose of propofol, mg/kg	1.5 ± 0.1	1.5 ± 0.1	0.089
Induction dose of sufentanil, µg	5.5 ± 1.0	5.6 ± 1.0	0.029
Total dose of propofol, mg	213.7 ± 48.4	212.5 ± 54.3	0.023
Baseline heart rate, beats/min	72.0 ± 9.2	73.7 ± 10.4	0.095
Baseline systolic blood pressure, mmHg	133.1 ± 9.8	133.8 ± 8.9	0.081
Baseline oxygen saturation, %	99.2 ± 0.9	99.4 ± 0.6	0.091

Data are presented as mean ± SD, median [IQR] or number (proportion). Abbreviations:SMD,Standardized Mean Difference; ASA, American Society of Anesthesiologists; BMI, body mass index; TMD, thyromental distance; EGD, esophagogastroduodenoscopy. STOP-BANG questionnaire score (Table S1 in Supplementary Appendix)

### Primary study outcome

Hypoxia (SpO_2_ < 90%, ≥ 10 s) occurred markedly less often in the capnography group than in the standard group (13.4% vs. 30.1%; *P* = 0.002) (Table 2).

**Table 2:** Study outcomes in the two groups

	Capnography (n = 112)	Standard (n = 113)	RR (95%CI)	*P* value
Primary study outcome				
Hypoxia (SpO_2_ < 90%,≥10 s)	15 (13.4)	34 (30.1)	0.44[0.26,0.77]	0.002
Secondary study outcomes				
Severe hypoxia (SpO_2_ ≤ 85%)	6 (5.4)	16 (14.2)	0.38[0.15,0.93]	0.026
Subclinical respiratory depression (90%≤SpO_2_ < 95%)	34 (30.4)	20 (17.7)	1.71[1.05,2.79]	0.026
SpO_2_ minimum	94.1 ± 5.9	92.7 ± 7.4		0.118
Conducting interventions	96 (85.7)	34 (30.1)	2.85[2.13,3.81]	0.000
Patient Satisfaction	4.9 ± 0.3	4.8 ± 0.3		0.479
Endoscopist Satisfaction (NAS, 1–10)	8.8 ± 1.1	8.6 ± 1.3		0.125
Anesthesia sedation adverse events				
PONV	3 (2.7)	0 (0)		0.242
Bradycardia (< 50 heartbeats/min)	6 (5.4)	7 (6.2)	0.86[0.30,2.49]	0.788
Hypotension (SBP < 90 mmHg)	1 (0.9)	1 (0.9)	1.01[0.06,15.93]	0.995
Premature ventricular contractions	2 (1.8)	3 (2.7)	0.67[0.12,3.95]	0.658
Management measures				
Ondansetron hydrochloride	3 (2.7)	0 (0)		0.242
Atropine	6 (5.4)	7 (6.2)	0.87[0.30,2.49]	0.788
Noradrenaline	1 (0.9)	1 (0.9)	1.01[0.06,15.93]	0.995
Lidocaine	2(1.8)	3 (2.7)	0.67[0.12,3.95]	0.658

Data are presented as mean ± SD, median [IQR] or number (proportion)

### Secondary study outcomes

The incidence of severe hypoxia was markedly lower in the capnography group (5.4% vs. 14.2%, *P* = 0.026) (Table 2). Subclinical respiratory depression was observed in 17.7% of the standard group but in 30.4% of the capnography group (*P* = 0.026) (Table 2).

**Fig. 2:**
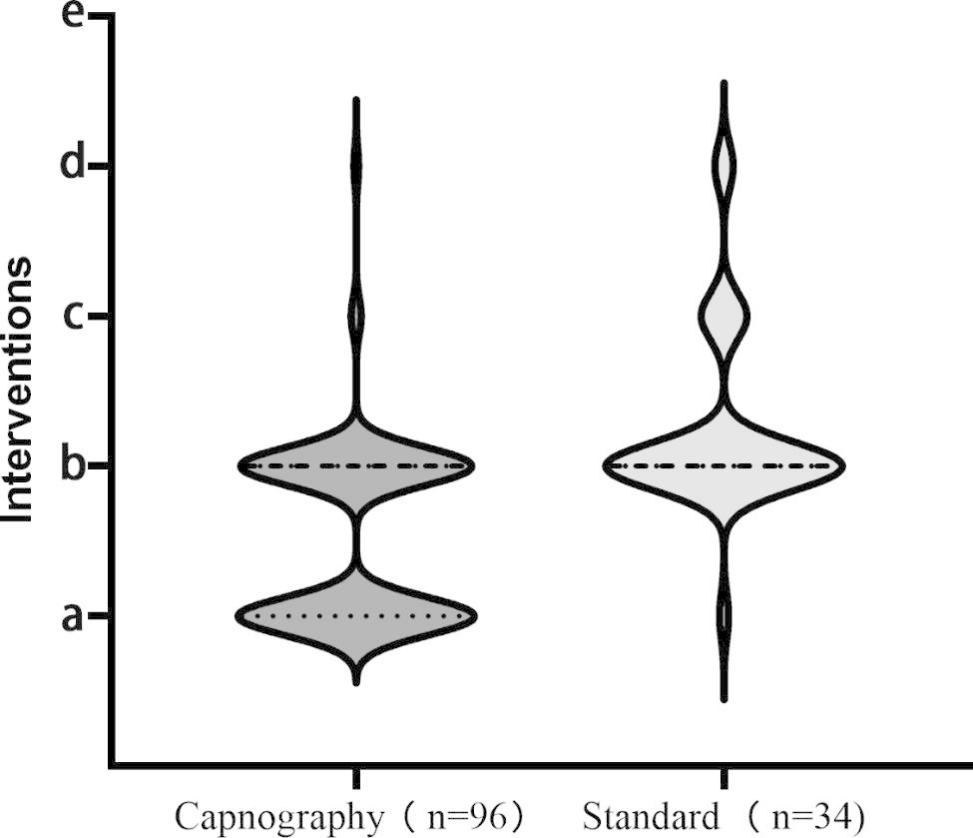
The violin plots show the frequency density of the five last interventions recorded during the sedation. In the event of inadequate alveolar ventilation during sedation, the investigators sequentially implemented five interventions until all respiratory-related parameters returned to normal. The five interventions are represented in alphabetical order(a-e): a: increasing oxygen flow (5 L/min), b: a chin lift or jaw thrust maneuvre, c: placement of the nasopharyngeal airway and chin lift, d: mask positive-pressure ventilation, and e: ventilator-assisted ventilation with tube insertion

In the capnography group, 30 and 66 patients developed altered ventilation and apnea, respectively. Thus, 96 (85.7%) patients underwent intervention in the capnography group(Fig. 2). Only 15 (13.4%) of these patients developed hypoxia after performing the intervention. None of the patients developed hypoxia without prior recording of apnoea or altered respiration. In the standard group, 34 (30.1%) of patients underwent intervention. There was a statistically significant difference (P < 0.0001) in the number of the last intraoperative intervention between the two groups (a:47 vs. 1, b:46 vs. 26, c:2 vs. 5, d:1 vs. 2, e:0 vs. 0) (Fig. 2).

When alveolar hypoventilation occurred during intravenous anesthesia, 47 (49%) of patients in the capnography group resolved their ventilation changes or airway abnormalities by increasing oxygen flow as opposed to only 1 (2.9%) patient in the standard group. Among the patients who received the intervention, 46 (47.9%) of the capnography group and 26 (76.5%) of the standard group had a chin lift or jaw thrust. Nasopharyngeal airways with jaw support were placed in 2 (2.1%) and 5 (14.7%) of patients in the capnography and standard groups, respectively. Two patients in the standard group and one in the capnography group underwent manual mask positive-pressure ventilation (Fig. 2, Table S6 in Supplementary Appendix).

The differences in minimum SpO_2_ during operation, patient satisfaction, or endoscopist satisfaction ratings between the two groups were not statistically significant (Table 2).

Adverse events of anesthesia sedation which occurred during the experiment, such as PONV, hypotension, bradycardia, and premature ventricular contractions, were treated with ondansetron hydrochloride, atropine, noradrenaline, and lidocaine, respectively. There was no statistically significant difference between the two groups (Table 2).

### Per-protocol analysis

A total of 225 patients were included in the intention-to-treat analysis. In per-protocol analysis, data are analyzed only for those patients who completely adhere to the treatment protocol. In the capnography group, two were excluded from the program analysis because they received midazolam and no oxygen supplementation. In the standard group, two patients were excluded from the program analysis because they received midazolam and etomidate instead of propofol. Thus, 221 participants were included in the per-protocol analysis. The results of statistical analysis for the per-protocol set were consistent with those of the intention-to-treat analysis. The results of the per-protocol analysis are reported in the Supplementary Material.

## Discussion

We investigated the effect of capnography on the incidence of hypoxia in mildly obese patients undergoing EGD and colonoscopy by sedation. We found that early intervention based on capnography reduced the incidence of hypoxia in patients and kept SpO_2_ in the subclinical respiratory depression stage in most patients. Capnography-based implementations increased the number of interventions for patients, but results show that nearly half of patients with alveolar hypoventilation in the capnography group improved oxygenation by increasing oxygen flow alone, whereas the standard group required further interventions.

There is a positive association between suppression of muscle activity and increased wet trapping of the upper airway with increasing doses of propofol [20]. This study shows that in most mildly obese patients, increasing oxygen flow through capnography in the early stages of ventilation changes or apnoea can prevent the patient from developing hypoxia; when interventions are made when SpO_2_ falls to 90%, the incidence of severe hypoxia increases. This delay may cause the patient to develop complete obstructive apnoea, where increasing oxygen flow is no longer effective and measures to open the airway (such as lifting the jaw) must be taken to improve full-body oxygenation. In a small number of obese patients with apnoea or a drop in SpO_2_, SpO_2_ did not improve even after a jaw lift, indicating central apnoea and necessity of special measures such as an oropharyngeal or nasopharyngeal ventilation tube with jaw lift and manual mask positive-pressure ventilation to improve oxygenation. Although additional capnography increases the intervention of patients and the intensity of the anesthesiologists’ work, nearly half of the mildly obese patients prevented hypoxia by increasing oxygen flow through early detection of hypopnea or apnoea, reducing the overall incidence of hypoxia.

Another randomized study [16] found that capnography during routine colonoscopy with propofol-based sedation reduced the rate of hypoxia in 533 adult patients. The mean BMI in this study was 25 kg/m^2^, and the incidence of hypoxia decreased from 32 to 18% (*P* < 0.001); in contrast, the mean BMI in our study was over 30 kg/m^2^ and the incidence of hypoxia decreased from 30.1 to 13.4% (*P* = 0.002). The differences in hypoxia incidence decrease may be due to non-uniformity of the sedation drug regimen in the opposing study and the administration of sedation by a combination of anesthetists, nurses, and endoscopists. Our study used a fixed-programmed sedation and analgesia model with a combination of sufentanil and propofol. The combination of propofol and an opioid (e.g., sufentanil) has a rapid onset of action and only causes mild respiratory depression; it is currently recommended for improved sedation and analgesia [21, 22]. Additionally, all sedation procedures were performed by anesthetists with extensive experience managing sedation-related complications.

Shao LJ et al. [23] performed supraglottic ventilation and oxygenation via a Weiss nasopharyngeal ventilation channel, which significantly reduced the incidence of hypoxia (SpO_2_ < 90%) in obese patients under propofol sedation for a gastroscopy (WNJT group: nasal cannula group = 8.1%: 38.5%). 6.1% of obese patients who underwent supraglottic ventilation and oxygenation via the Weiss nasopharyngeal airway developed nasal bleeding. In contrast, real-time capnographic monitoring of the ventilation of obese patients in our study did not interfere with normal nasal cannula oxygenation, and intervention based on capnographic waveform maps did not causing complications such as nasal bleeding.

A prospective cohort study [24] of 82 morbidly obese patients with a mean BMI of 46.4 ± 8.2 kg/m^2^ who underwent capnographic monitoring during gastroscopy under propofol sedation showed that abnormal P_ET_CO_2_ marked the onset of all respiratory depression, as determined by a decrease in P_ET_CO_2_ of > 10 mmHg from baseline or the disappearance of the P_ET_CO_2_ waveform. The sensitivity and predictive value of this depression was 81% and 78%, respectively. The capnography in this study detected oral exhaled CO_2_; however, the authors indicated that six patients developed nasal breathing after sedation, which may have lowered the monitoring accuracy. The capnography device (capnostream-20P) used in our study allowed simultaneous monitoring of both nasal and transoral exhaled CO_2_; a pump extracted a small portion of the exhaled gas directly to an infrared detection probe. The device was easily applied to non-intubated patients, and provided a small volume of sampled gas which could be measured with high sensitivity and fast response.

Our study has some limitations. Firstly, the depth of sedation using the bispectral index (BIS) was not recorded during the study. In theory, more profound sedation would increase how hypoxic the episodes in subjects would be. Chen et al. demonstrated a considerable lag between the BIS and MOAA/S scores, especially during the initial titration and recovery from sedation [25]. In addition, the guideline [26] do not recommend using BIS as a routine monitoring method for endoscopy. Patients in both groups were well sedated after the administration of propofol and sufentanil, and none of the patients were prematurely terminated due to inadequate sedation. We believe that the randomization process resulted in a fair distribution of episodes of deep sedation between the two groups; therefore, recording the depth of sedation did not significantly impact the overall study results. Despite this, knowing the depth of sedation may improve our understanding of hypoxia, which can be caused by deep sedation. Secondly, to compare the effectiveness of capnography with SpO_2_ monitoring of patients’ ventilation status, intervention was started only when SpO_2_ in the standard group dropped below 90% during the study. Intervention was not performed early in high-risk patients with morbid obesity, which may have caused serious adverse outcomes for the patients. In our study, none of the patients experienced serious adverse outcomes, such as accidental hospitalization, permanent neurological damage, aspiration pneumonia, or death. Finally, we did not include patients with ASA class III, who often have a lack of respiratory reserve and can suffer a significant impact from apnea. Because obesity was a major factor in our study, we intend to continue our study in patients with ASA class III or higher in the future.

## Conclusion

In conclusion, capnography provides a near real-time ventilation assessment in patients with preserved autonomic breathing and is an excellent tool for monitoring transient hypoxia in sedated patients. Our study shows that additional capnography in a group of mildly obese patients prone to hypoxia effectively reduces the incidence of hypoxia and severe hypoxia.

## Electronic supplementary material

Below is the link to the electronic supplementary material.

Supplementary Appendix

## Data Availability

The datasets generated and analysed during the current study are not publicly available due to institutional restrictions but are available from the corresponding author on reasonable request.

## Abbreviations

EGD: Esophagogastroduodenoscopy
SpO_2_: Pulse oxygen saturation
P_ET_CO_2_: Postapneic end-tidal carbon dioxide
BMI: Body mass index
ASA: American Society of Anesthesiologists
ChiCTR: Chinese Clinical Trial Registry
SAE: Severe adverse event
MOAA/S: Modified Observer’s Assessment of Alertness/Sedation
PADSS: Post anesthesia discharge scoring system
PONV: Postoperative nausea and vomiting
OSA: Obstructive sleep apnea
BIS: Bispectral index
MAP: Mean arterial pressure
HR: Heart rate

## References

[1] Wu Z, Yu J, Zhang T, Tan H, Li H, Xie L (2022). Effects of Etco2 on the Minimum Alveolar concentration of sevoflurane that blunts the adrenergic response to Surgical incision: a prospective, randomized, double-blinded trial. Anesth Analg.

[2] Practice guidelines for (2002). Sedation and analgesia by non-anesthesiologists. Anesthesiology.

[3] Dumonceau JM, Riphaus A, Aparicio JR, Beilenhoff U, Knape JT, Ortmann M (2010). European Society of Gastrointestinal Endoscopy, European Society of Gastroenterology and Endoscopy Nurses and Associates, and the European Society of Anesthesiology Guideline: non-anesthesiologist administration of propofol for GI endoscopy. Endoscopy.

[4] Qadeer MA, Lopez AR, Dumot JA, Vargo JJ (2011). Hypoxemia during moderate sedation for gastrointestinal endoscopy: causes and associations. Digestion.

[5] Xiao Q, Yang Y, Zhou Y, Guo Y, Ao X, Han R (2016). Comparison of nasopharyngeal Airway device and nasal oxygen tube in obese patients undergoing intravenous anesthesia for Gastroscopy: a prospective and randomized study. Gastroenterol Res Pract.

[6] Zavorsky GS, Hoffman SL (2008). Pulmonary gas exchange in the morbidly obese. Obes Rev.

[7] Steier J, Lunt A, Hart N, Polkey MI, Moxham J (2014). Observational study of the effect of obesity on lung volumes. Thorax.

[8] Vrishali RA, Manish P, Naresh GT. Neck circumference to thyromental distance ratio: is a reliable predictor of difficult intubation in obese patients. Indian J Clin Anaesth. 2019;6(1).

[9] Qadeer Mohammed A, Rocio Lopez A, Dumot John A, Vargo John J. Risk factors for hypoxemia during ambulatory gastrointestinal endoscopy in ASA I-II patients. Dig Dis Sci. 2009;54(5). 10.1007/s10620-008-0452-2 19003534

[10] Wani S, Azar R, Hovis CE, Hovis RM, Cote GA, Hall M (2011). Obesity as a risk factor for sedation-related complications during propofol-mediated sedation for advanced endoscopic procedures. Gastrointest Endosc.

[11] Mehta PP, Kochhar G, Kalra S, Maurer W, Tetzlaff J, Singh G (2014). Can a validated sleep apnea scoring system predict cardiopulmonary events using propofol sedation for routine EGD or colonoscopy? A prospective cohort study. Gastrointest Endosc.

[12] Mehta PP, Kochhar G, Albeldawi M, Kirsh B, Rizk M, Putka B (2016). Capnographic Monitoring in Routine EGD and Colonoscopy with Moderate Sedation: a prospective, randomized, controlled trial. Am J Gastroenterol.

[13] Vargo JJ, Zuccaro G, Dumot JA, Conwell DL, Morrow JB, Shay SS (2002). Automated graphic assessment of respiratory activity is superior to pulse oximetry and visual assessment for the detection of early respiratory depression during therapeutic upper endoscopy. Gastrointest Endosc.

[14] Burton JH, Harrah JD, Germann CA, Dillon DC (2006). Does end-tidal carbon dioxide monitoring detect respiratory events prior to current sedation monitoring practices. Acad Emerg Med.

[15] Beitz A, Riphaus A, Meining A, Kronshage T, Geist C, Wagenpfeil S (2012). Capnographic monitoring reduces the incidence of arterial oxygen desaturation and hypoxemia during propofol sedation for colonoscopy: a randomized, controlled study (ColoCap Study). Am J Gastroenterol.

[16] Friedrich-Rust M, Welte M, Welte C, Albert J, Meckbach Y, Herrmann E (2014). Capnographic monitoring of propofol-based sedation during colonoscopy. Endoscopy.

[17] Zongming J, Zhonghua C, Xiangming F (2014). Sidestream capnographic monitoring reduces the incidence of arterial oxygen desaturation during propofol ambulatory anesthesia for surgical abortion. Med Sci Monit.

[18] Barnett S, Hung A, Tsao R, Sheehan J, Bukoye B, Sheth SG (2016). Capnographic Monitoring of Moderate Sedation during Low-Risk Screening Colonoscopy does not improve safety or patient satisfaction: a prospective cohort study. Am J Gastroenterol.

[19] Chernik DA, Gillings D, Laine H, Hendler J, Silver JM, Davidson AB (1990). Validity and reliability of the Observer·s Assessment of Alertness/Sedation scale: study with intravenous midazolam. J Clin Psychopharmacol.

[20] Hillman DR, Walsh JH, Maddison KJ, Platt PR, Kirkness JP, Noffsinger WJ (2009). Evolution of changes in upper airway collapsibility during slow induction of anesthesia with propofol. Anesthesiology.

[21] Zhao YJ, Liu S, Mao QX, Ge HJ, Wang Y, Huang BQ (2015). Efficacy and safety of remifentanil and sulfentanyl in painless gastroscopic examination: a prospective study. Surg Laparosc Endosc Percutan Tech.

[22] Yin N, Xia J, Cao YZ, Lu X, Yuan J, Xie J (2017). Effect of propofol combined with opioids on cough reflex suppression in gastroscopy: study protocol for a double-blind randomized controlled trial. BMJ Open.

[23] Shao LJ, Hong FX, Liu FK, Wan L, Xue FS (2021). Prospective, randomized comparison of two supplemental oxygen methods during gastroscopy with propofol mono-sedation in obese patients. World J Clin Cases.

[24] Prathanvanich P, Chand B (2015). The role of capnography during upper endoscopy in morbidly obese patients: a prospective study. Surg Obes Relat Dis.

[25] Chen SC, Rex DK (2004). An initial investigation of bispectral monitoring as an adjunct to nurse-administered propofol sedation for colonoscopy. Am J Gastroenterol.

[26] Early DS, Lightdale JR, Vargo JJ, Acosta RD, Chandrasekhara V, Chathadi KV (2018). Guidelines for sedation and anesthesia in GI endoscopy. Gastrointest Endosc.

