# Supplementary material for: The effect of capnography on the incidence of hypoxia during sedation for EGD and colonoscopy in mildly obese patients: a randomized, controlled study
Source: BMC Anesthesiol. 2023 May 31;23:188. doi: 10.1186/s12871-023-02151-8 (PMC10230763; doi:10.1186/s12871-023-02151-8)
Supplement: Supplementary file 1 — Supplementary Appendix [file 12871_2023_2151_MOESM1_ESM.pdf]

Supplementary Appendix

This appendix has been provided by the authors to give readers additional information about their work.

Supplementary Tables

Table S1. STOP-BANG questions and scoringa (From Jirapinyo and Thompson [1], with permission)

S	Snoring: Do you snore loudly (louder than talking or loud enough to be heard through closed doors)?	Yes/no
T	Tired: Do you often feel tired, fatigued, or sleepy during the daytime	Yes/no
O	Observed: Has anyone observed you stop breathing during your sleep?	Yes/no
P	Blood pressure: Have you been or are you being treated for high blood pressure?	Yes/no
B	BMI: >35 kg/m^2	Yes/no
A	Age: Older than 50 years	Yes/no
N	Neck circumference: >40 cm	Yes/no
G	Gender: Male	Yes/no

[a]High risk of OSA: yes to three or more questions; low risk of OSA: yes to less than three questions.

Table S2. The modified Observer's Assessment of Alertness/Sedation（MOAA/S ） Scale

MOAA/S Scale	Scale	ASA Classifification
Responds readily to name spoken in normal tone	5 (alert)	Minimal
Lethargic response to name spoken in normal tone	4	Moderate
Responds only after name is called loudly and/or repeatedly	3	Moderate
Responds only after mild prodding or shaking	2	Moderate
Responds only after painful trapezius squeeze	1	Deep
Does not respond to painful trapezius squeeze	0	Deep/general anesthesia

ASA: American Society of Anesthesiologists.

Table S3. Post anesthetic discharge scoring system (PADSS)

criteria Metrics	score	criteria
vital signs	2	Fluctuation within 20% of preoperative level
	1	Within 10% to 40% of preoperative level
	0	40% of preoperative value
ambulation	2	Orientation and steady gait
	1	Orientation or steady gait
	0	Able to achieve either of the first two
nausea/vomiting	2	Very few
	1	Medium
	0	Severe
pain	2	Very few
	1	Medium
	0	Severe
bleeding	2	Very few
	1	Medium
	0	Severe

10 out of 10, score of ≥9 for discharge

Table S4. Patient satisfaction with the sedation

score	criteria
5	excellent, no pain
4	good, mild pain
3	fair, much discomfort but tolerable
2	poor, very uncomfortable and painful
1	very poor, very painful to the point of being unbearable

Table S5. Anesthesia sedation adverse event record

Step 1：Any adverse events?	
□ NO	□ YES（Continue to fill out the table below）

Step 2: Describe the adverse event		
Mild hazard event description	Moderate risk event description	Alert Hazardous Event Description
● Nausea, vomiting<br>● Muscle stiffness, clonus<br>● Excessive oral secretions<br>● Anomalous reaction*1<br>● Restlessness during awakening*2<br>● Extended awakening time*3	● Sedation failure*4<br>● Non-allergic rash<br>● Bradycardia*5<br>● Tachycardia*5<br>● Hypertension*5<br>● Hypotension*5<br>● Epilepsy<br>● Allergic reactions	● Circulatory collapse/shock*6<br>● Cardiac arrest/pulseless<br>● Anaphylactic shock<br>● Ventricular arrhythmias<br>● Regurgitation and malabsorption

Step 3: Handling adverse events			
Mild hazard event description	Moderate risk event description	Alert Hazardous Event Description	Others
● No treatment<br>● Tactile stimulation<br>● Application of anti-salivary medication<br>● Supplemental sedation<br>● Application of antiemetic<br>● Application of antihistamines	● Mask ventilation<br>● Placement in the nasopharyngeal/oropharyngeal airway<br>● Laryngeal Mask Ventilation<br>● Continuous Positive Airway Pressure Ventilation<br>● Application of pro-arousal drugs, including flumazenil, naloxone, etc.<br>● Rapid intravenous infusion<br>● Application of anticonvulsant and antiepileptic drugs<br>● Application of cardiovascular active drugs,including atripine epinephrine,etc	● External chest compressions<br>● Glucocorticoids<br>● Lidocaine<br>● Application of vasoactive drugs such as atropine and epinephrine	
**Step 4: Patient prognosis**			
Prognosis of mild risk events	Prognosis of moderate risk events	Prognosis of alert hazardous events	Others

● No adverse prognosis	● Accidental hospitalization<br>● Medical Upgrades*6	●Patient death<br>● Permanent neurological deficits<br>Aspiration pneumonia●*7	

Description:

*1Restlessness during awakening：Unanticipated restlessness and uneasiness about sedation

*2Restlessness during awakening：Abnormal reactions during patient recovery, including crying, irritability, delirium, hallucinations, and nightmares

*3Extended awakening time：No awakening to preoperative level within 2 hours

*4 Sedation failure：No way to keep the patient in a stable state for a successful surgery

*5 Circulatory collapse/shock：Inadequate perfusion with clinical evidence

*6 Medical Upgrades：Patients are admitted to the ICU from a general ward, or have an extended hospital stay

*7 Aspiration pneumonia：Suspected inhalation of exogenous substances, including gastric contents, and associated with new or worsening pulmonary manifestations

Table S6. The last interventions during the procedure

	Capnography (n=96)	Standard （n=34）	*P*
Interventions,n(%)			0.000
Increasing oxygen flow (5 L/min)	47(49.0)	1(2.9)	
A chin lift or jaw thrust maneuver	46(47.9)	26(76.5)	
Placement of the nasopharyngeal airway and chin lift	2(2.1)	5(14.7)	
Mask positive-pressure ventilation	1(1.0)	2(5.9)	
Ventilator-assisted ventilation with tube insertion	0	0	

STATISTICAL ANALYSES

Per Protocol analysis

Table S7. Demographic, clinical, and procedural characteristics of subjects — per-protocol analysis

	Standard （n=111）	Capnography （n=110）	*P*
Age (years)	48.50[38.75 to 55.2]	46.50[37.7 to 56.2]	0.860
Sex, male	86（77.5）	83（75.5）	0.723
BMI ,kg/m^2	30.45[29.3 to 31.8]	30.09[28.9 to 32.4]	0.550
ASA class			0.296
I	93(82.9)	85(77.3)	
II	19 (17.1)	25(22.7)	
History of smoking	37（33.3）	41（37.3）	0.540
History of alcohol consumption	38（34.2）	40（36.4）	0.740
Mallampati Class			0.544
I	38(34.2)	34(30.9)	
II	70(63.1)	70(63.6)	
III	3(2.7)	6(5.5)	
TMD＜6cm	24（21.6）	27（24.5）	0.606
Micrognathia or retrognathia	16（14.4）	16（14.5）	0.978
STOP-BANG questionnaire score ＞3 分	34（30.6）	34（30.9）	0.964
Type of procedure			0.462
EGD	43（38.7）	41（37.3）	
Colonoscopy	13（11.7）	8（7.3）	
Colonoscopy + EGD	55（49.5）	61（55.5）	
Procedure time, minutes	25[14 to 33]	25[15 to 33]	0.812
Induction dose of propofol, mg/Kg	1.5[1.4 to 1.5]	1.5[1.4 to 1.5]	0.632
Induction dose of Sufentanil, ug	5.0[0.0 to 0.0]	5.0[0.0 to 0.0]	0.831
Total dose of propofol, mg	200[170 to 245]	210[180 to 250]	0.562
Polypectomy	13（11.7）	21（19.1）	0.128
Baseline heart rate, beats/min	72.5[66 to 80]	70[67 to 77]	0.304
Baseline systolic blood pressure, mmHg	132[128 to 140]	132[127 to 137]	0.508
Baseline oxygen	99[99 to 100]	99[99 to 100]	0.186

saturation, %

Data are presented as medians [IQR] or numbers (proportions). ASA, American Society of Anesthesiologists; BMI, body mass index; TMD, thyromental distance; EGD, esophagogastroduodenoscopy.

Table S8. Preselected outcomes in the two groups — per-protocol analysis

	Standard （n=111）	Capnography （n=110）	*P*
Hypoxia （SpO_2＜90%,≥10s）	34（30.6）	15（13.6）	0.002
Severe hypoxia （SpO_2 ≤ 85%）,	16（14.4）	6（5.5）	0.026
Subclinical respiratory depression （90%≤SpO_2< 95%）	20（18）	34（30.9）	0.026
SpO_2 minimum	95[88 to 98]	95[92 to 97]	0.581
Conducting interventions	34（30.1）	94（85.5）	0.000
Patient Satisfaction	5[5 to 5]	5[5 to 5]	0.364
Endoscopist Satisfaction (NAS, 1 – 10)	9[8 to 10]	9[8 to 10]	0.295

Data are presented as medians [IQR] or numbers (proportions). SBP, systolic blood pressure.

Table S9. Anesthesia sedation adverse events and management measures—per-protocol analysis

	Standard （n=111）	Capnography （n=110）	*P*
Anesthesia sedation adverse events			
PONV	0（0）	3（2.7）	0.242
Bradycardia(＜50 heartbeats/min)	7（6.3）	5（4.5）	0.768
Hypotension (BP < 90 mmHg)	1（0.9）	1（0.9）	0.998
Premature intraoperative ventricular contractions	3（2.7）	2（1.8）	0.549
Management measures			
Ondansetron hydrochloride	0（0）	3（2.7）	0.242
Atropine	7（6.3）	5（4.5）	0.768
Noradrenaline	1（0.9）	1（0.9）	0.998
Lidocaine	3（2.7）	2（1.8）	0.549

Data are presented as medians [IQR] or numbers (proportions). PONV, Post-operative nausea and vomiting.

Table S10. Intraoperative interventions used for corrective ventilation— per-protocol analysis

	Standard（n=111）	Capnography（n=110）	*P*
Interventions			0.000
Increasing oxygen flow(5L/min)	1（2.9）	45（47.9）	
A chin lift or jaw thrust maneuver	26（76.5）	46（48.9）	
Placement of the nasopharyngeal airway and a chin lift	5（14.7）	2（2.1）	
Artificial mask positive pressure ventilation	2（5.9）	1（1.1）	

References

[1] P Jirapinyo CCT. Sedation challenges: obesity and sleep apnea. Gastrointestinal Endoscopy. 2016 .
